# Venous thromboembolism and transfusion outcomes following intravenous tranexamic acid in primary total hip arthroplasty: A prospective two-center cohort study

**DOI:** 10.1051/sicotj/2026068

**Published:** 2026-11-13

**Authors:** Jairo Alonso Rincon-Hoyos, David Rincon, Jorge Manrique, Andres Restrepo, Jorge Casas, Sofia Munoz-Medina, Alejandro Torres-Mayorga

**Affiliations:** 1 GIOT Research Group, Department of Orthopedics and Traumatology, Clínica Colsanitas, Clínica Universitaria Colombia Carrera. 66 #23–46 Bogotá Bogotá D.C Colombia; 2 Department of Orthopedics and Traumatology, Clinica Universitaria Colombia Cra. 66 #23–46 Bogotá Bogotá D.C Colombia; 3 GIOT Research Group, Research Unit Fundación Universitaria Sanitas Cl. 170 #8–41 Bogotá Bogotá D.C Colombia; 4 GIOT Research Group, Department of Orthopedics and Traumatology, Clínica Colsanitas, Fundación Universitaria Sanitas, Clínica Universitaria Colombia Cra. 66 #23 46 Bogotá Bogotá D.C Colombia

**Keywords:** Tranexamic acid, Total hip arthroplasty, Venous thromboembolism, Deep vein thrombosis, Blood transfusion, Blood conservation

## Abstract

*Introduction*: Tranexamic acid (TXA) is widely used during total hip arthroplasty (THA) to reduce perioperative blood loss and transfusion requirements. Although extensive evidence supports its use, prospective real-world data from Latin America are limited. This study aimed to describe the incidence of symptomatic venous thromboembolism (VTE) and blood transfusion among patients receiving intravenous TXA during primary total hip arthroplasty (THA) at two institutions in Bogotá, Colombia. *Methods*: This prospective, observational, descriptive, two-center cohort study included consecutive adults who underwent elective primary THA. Patients received intravenous TXA according to institutional protocols. The primary outcome was the occurrence of symptomatic VTE, including deep vein thrombosis (DVT), pulmonary embolism, ischemic stroke, and myocardial infarction during a 10-week follow-up period. The secondary outcomes included blood transfusion requirements, postoperative hemoglobin levels, and emergency department visits. *Results*: A total of 151 primary THAs were performed in 133 patients. One case of symptomatic DVT was diagnosed, corresponding to a VTE incidence of 0.75%. Five patients (3.8%) required postoperative blood transfusions, including two complex reconstructive procedures. The mean postoperative hemoglobin level was 11.8 ± 1.9 g/dL. No pulmonary embolism, ischemic stroke, or myocardial infarction was observed during the follow-up period. *Conclusions*: In this prospective two-center cohort of patients receiving intravenous TXA during primary THA, including both conventional and complex cases, a low incidence of symptomatic VTE and transfusion was observed. These results provide prospective real-world evidence from a Latin American population and contribute to the regional literature on perioperative blood conservation strategies for primary THA.

## Introduction

Total hip arthroplasty (THA) is one of the most successful and frequently performed orthopedic procedures worldwide. [1, 2] In 2020, approximately 655,000 THAs were performed in the United States, with a projected 284% increase by 2040 [3].

The rising number of THAs has increased interest in reducing perioperative complications. Postoperative blood loss after THA remains a major cause of anemia, allogeneic blood transfusion, prolonged hospital stay, delayed rehabilitation, and greater healthcare resource use [1, 4–8].

In joint replacement surgery, blood loss may exceed 1,300 mL, with about 90% occurring during the first 24 h [1, 9]. Reported blood transfusion rates following THA range from 21% to 70% [10, 11]. Accordingly, blood-conservation strategies, including pharmacological agents, are integral to contemporary total joint arthroplasty protocols [10].

Tranexamic acid (TXA) is a synthetic amino acid with antifibrinolytic activity that inhibits fibrin degradation and reduces perioperative blood loss. [7, 12] TXA is widely used in total joint arthroplasty because it reduces blood loss and transfusion requirements [1, 4, 6–8, 13, 14].

Although isolated reports have described complications associated with TXA, including venous thromboembolism (VTE), renal failure, increased inflammatory markers, and chondrotoxicity after topical application [15–18], evidence from randomized trials, meta-analyses, and guidelines supports its routine use without increased symptomatic VTE or other major thromboembolic complications when used appropriately [6, 10, 19].

TXA has also been reported safe in patients with severe systemic disease (ASA III–IV), without increased VTE within the first 30 postoperative days after arthroplasty [20].

Despite extensive support for TXA use, most evidence comes from trials or registry studies in North America, Europe, and Asia, while prospective observational data from Latin America remain limited. At the participating institutions, intravenous TXA is part of standardized perioperative blood-conservation protocols for primary and revision THA. Therefore, this prospective, observational, two-center cohort study aimed to describe the incidence of symptomatic VTE after routine intravenous TXA administration in patients undergoing primary THA.

By reporting prospective real-world data from two orthopedic referral centers in Colombia, this study contributes regional evidence to the growing body of literature evaluating perioperative blood-conservation strategies in total hip arthroplasty.

## Materials and methods

This prospective, observational, descriptive, two-center cohort study included consecutive adult patients (≥18years) who underwent elective primary THA between August 1, 2019, and January 30, 2020, at two orthopedic referral centers in Bogotá, Colombia. The study was approved by the Institutional Ethics Committee (Protocol No. 033-19/08-2019) and conducted in accordance with the Declaration of Helsinki and applicable national regulations. Written informed consent was obtained from all participants before enrollment.

Consecutive eligible patients were recruited throughout the predefined study period. Both conventional primary THA and complex primary reconstructive procedures were included to reflect the routine case mix managed at these referral centers. Complex procedures comprised severe developmental dysplasia of the hip, chronic hip dislocation, and conversion THA following previous internal fixation. Patients were excluded if they underwent revision THA; had blood dyscrasias, severe hepatic disease, chronic kidney disease (estimated glomerular filtration rate <30 mL/min/1.73 m^2^), epilepsy or previous seizures, known hypersensitivity to TXA; or declined participation.

All procedures were performed through a posterolateral approach by three fellowship-trained hip surgeons following a standardized institutional protocol. Intravenous TXA was administered as 1 g within 30 min before skin incision and 1 g approximately 3 h after surgery. Perioperative antibiotic prophylaxis consisted of cefazolin or clindamycin in patients with β-lactam allergy. Mechanical thromboprophylaxis included intraoperative intermittent pneumatic compression, while pharmacological prophylaxis consisted of enoxaparin followed by acetylsalicylic acid after 10 days to complete 30 days of thromboprophylaxis.

Postoperative hemoglobin and hematocrit were routinely measured approximately 8 h after surgery. Rehabilitation was initiated under physiotherapist supervision on the first postoperative day for hospitalized patients or before discharge in the outpatient pathway. Hospitalized patients were discharged on the first postoperative day whenever clinically appropriate, whereas outpatient arthroplasty patients were discharged approximately 6 h after surgery after meeting standardized discharge criteria and received home nursing supervision for the first 72 postoperative hours.

Patients attended routine outpatient evaluation approximately one week after surgery and were prospectively followed for 10 weeks, including standardized telephone follow-up to identify postoperative complications and emergency department visits. The primary outcome was the occurrence of symptomatic venous thromboembolism (deep vein thrombosis, pulmonary embolism, ischemic stroke, or myocardial infarction). Secondary outcomes included postoperative blood transfusion, postoperative hemoglobin concentration, and emergency department visits. Baseline variables included age, sex, body mass index, American Society of Anesthesiologists (ASA) physical status, comorbidities, previous thromboembolic or ischemic disease, chronic antithrombotic therapy, procedure laterality, unilateral or staged bilateral THA, and adherence to the institutional TXA protocol.

VTE surveillance was symptom-driven. Patients with suspected DVT underwent venous Doppler ultrasonography, whereas pulmonary embolism and other thromboembolic events were investigated according to routine clinical practice. Consequently, asymptomatic VTE may not have been detected. Blood transfusion was administered according to institutional protocols, using a hemoglobin threshold of 8 g/dL in symptomatic patients. Estimated intraoperative blood loss, calculated blood loss, hidden blood loss, intraoperative hemoglobin values, and perioperative hemoglobin changes were not prospectively collected.

## Statistical analysis

Continuous variables are presented as mean ± standard deviation (SD) or median (interquartile range [IQR]), as appropriate. Categorical variables are reported as frequencies and percentages. Comparisons between first- and second-stage procedures among patients undergoing staged bilateral THA were performed using Student’s *t*-test or the Wilcoxon rank-sum test for continuous variables and Pearson’s chi-square test or Fisher’s exact test for categorical variables, as appropriate. A two-sided *p* < 0.05 was considered statistically significant. Statistical analyses were performed using IBM SPSS Statistics for Windows, Version 22.0 (IBM Corp., Armonk, NY, USA).

## Results

Between August 1, 2019, and January 30, 2020, 151 primary THA procedures were performed in 133 consecutive patients at the two participating institutions. Eighteen patients underwent staged bilateral THA (36 procedures, 23.8%), whereas 115 underwent unilateral THA (76.2%).

The study population comprised 93 women (61.6%) and 58 men (38.4%), with a median age of 60 years (IQR 19). Most patients were classified as ASA II (67.5%), and seven had a history of thromboembolic or ischemic disease. Baseline demographic and clinical characteristics are summarized in Table 1.

**Table 1:** Baseline demographic and clinical characteristics of the study population.

Characteristic	Value
Patients, *n*	133
Age, years, median (IQR)	60 (19)
Female sex, *n* (%)	93 (61.6)
Male sex, *n* (%)	58 (38.4)
BMI, kg/m^2^, mean ± SD	26.6 ± 4.8
ASA classification, *n* (%)	
ASA I	25 (16.5)
ASA II	102 (67.5)
ASA III	24 (15.9)
Previous thromboembolic/ischemic disease, *n* (%)	
Deep vein thrombosis	1 (0.7)
Pulmonary embolism	1 (0.7)
Ischemic cardiomyopathy	5 (3.3)
Ischemic stroke	1 (0.7)
Comorbidities, *n* (%)	
Hypertension	34 (22.5)
Hypothyroidism	23 (15.2)
Dyslipidemia	20 (13.2)
Diabetes mellitus	13 (8.6)
Rheumatoid arthritis	6 (4.0)
Chronic obstructive pulmonary disease	2 (1.3)
Chronic kidney disease	2 (1.3)
Previous cancer	1 (0.7)
Myasthenia gravis	1 (0.7)
Chronic antithrombotic therapy, *n* (%)	
Aspirin	3 (2.0)
Clopidogrel	2 (1.3)
Enoxaparin	1 (0.7)
Direct oral anticoagulant	1 (0.7)

BMI, body mass index; ASA, American Society of Anesthesiologists; SD, standard deviation; IQR, interquartile range.

All patients received at least one intravenous dose of TXA. A preoperative dose was administered in 139 procedures (92.0%) and a postoperative dose in 135 (89.4%). Complete adherence to the two-dose protocol was achieved in 123 procedures (81.5%), whereas 28 procedures (18.5%) received only one dose because of deviations from routine clinical practice. No significant differences were observed between the first- and second-stage bilateral procedures regarding TXA administration, postoperative hemoglobin concentration, transfusion requirement, or emergency department visits. First-stage procedures were more frequently performed on an outpatient basis (*p* < 0.001). Surgical characteristics are presented in Table 2.

**Table 2:** Perioperative characteristics and clinical outcomes of the 151 primary THA procedures.

Variable	Value
Procedures, *n*	151
Institution, *n* (%)	
Institution A	87 (57.6)
Institution B	64 (42.4)
Laterality, *n* (%)	
Right	89 (58.9)
Left	62 (41.1)
Procedure type, *n* (%)	
Unilateral THA	115 (76.2)
Staged bilateral THA	36 (23.8)
Fixation, *n* (%)	
Uncemented	150 (99.3)
Hybrid	1 (0.7)
TXA administration, *n* (%)	
Preoperative dose	139 (92.0)
Postoperative dose	135 (89.4)
Both doses	123 (81.5)
One dose only	28 (18.5)
Clinical outcomes, *n* (%)	
Symptomatic VTE (Patient level, *n* = 133)	1 (0.75)
Blood transfusion (Patient level, *n* = 133)	5 (3.8)
Emergency department visit	18 (12.0)
Outpatient management	92 (60.9)
Hospital admission	59 (39.1)
Postoperative hemoglobin, mean ± SD (g/dL)	11.8 ± 1.9

Patients were prospectively followed for 10 weeks. One symptomatic DVT occurred one week after surgery, corresponding to a symptomatic VTE incidence of 0.75%. No pulmonary embolism, ischemic stroke, or myocardial infarction was observed. The affected patient was a 67-year-old woman (ASA III) with obesity (BMI 37.6 kg/m^2^), a previous history of DVT and pulmonary embolism, and multiple cardiovascular comorbidities who underwent unilateral cementless primary THA after receiving both scheduled TXA doses.

The mean postoperative hemoglobin concentration was 11.8 ± 1.9 g/dL. Five patients (3.8%) required postoperative blood transfusion according to institutional criteria; two followed unilateral THA and three followed the second stage of staged bilateral THA. Two of the latter corresponded to complex reconstructive procedures (Crowe type IV developmental dysplasia of the hip and bilateral conversion THA after previous osteosynthesis).

During follow-up, 18 patients (12.0%) visited the emergency department, most commonly because of postoperative pain (33.3%) or prosthetic dislocation (11.1%). Emergency department visits and postoperative outcomes are summarized in Table 3.

**Table 3:** Reasons for emergency department visits.

Reason	*n* (%)
Pain	6 (33.3)
Implant dislocation	2 (11.1)
Superficial wound infection	2 (11.1)
Falls	2 (11.1)
Nausea/vomiting/dizziness	2 (11.1)
Fever	1 (5.6)
Wound dehiscence	1 (5.6)
Symptomatic VTE	1 (5.6)
Periprosthetic fracture	1 (5.6)

## Discussion

This prospective, two-center, observational cohort study describes the incidence of symptomatic VTE and blood transfusion among patients undergoing primary THA managed with routine intravenous TXA. During the 10-week follow-up, symptomatic VTE (0.75%) and transfusion (3.8%) rates were low. These findings provide prospective real-world data from two orthopedic referral centers in Colombia and complement existing evidence regarding routine TXA use in primary THA.

The role of TXA in reducing perioperative blood loss during total joint arthroplasty is well established [8, 19]. Multiple randomized controlled trials, meta-analyses, and international clinical practice guidelines have consistently demonstrated reduced blood loss and transfusion requirements with TXA administration [21, 22].

TXA administration protocols remain heterogeneous [1]. Wilde et al. [23] analyzed 1,736 THAs and reported a smaller postoperative hemoglobin decrease (2.9 vs. 3.6 g/dL), transfusion rates of 0.7% with two TXA doses, no transfusions with one dose, and 12.5% without TXA. Similarly, Akti et al. [4] found significantly lower transfusion requirements in patients receiving TXA.

The overall transfusion rate observed in this cohort (3.8%) was comparable with the rates reported in contemporary series of primary THA using intravenous TXA. Although direct comparisons should be interpreted cautiously because of differences in patient selection, surgical complexity, transfusion thresholds, and TXA protocols, our findings are consistent with the growing body of literature reporting low transfusion requirements following routine antifibrinolytic use. Notably, three of the five transfusions occurred after the second stage of staged bilateral THA, and two of these patients underwent complex reconstructive procedures involving Crowe type IV developmental dysplasia or conversion THA after previous osteosynthesis procedures.

Complex primary THA, including developmental dysplasia, chronic hip dislocation, conversion THA, and staged bilateral procedures, was intentionally retained because these represent routine practice at the participating referral centers. Although these procedures carry greater bleeding risk, the small number of outcome events precluded meaningful subgroup or sensitivity analyses. Accordingly, the study was designed to describe outcomes across the full spectrum of primary THA rather than compare procedural complexity.

One symptomatic DVT occurred (0.75%). The patient had obesity (BMI 37.6), previous DVT and PE, hypertension, dyslipidemia, hypothyroidism, and chronic clopidogrel therapy. Previous VTE, obesity, hypertension, and dyslipidemia are recognized VTE risk factors [24, 25].

Likewise, Whiting et al. [20] did not find an increase in the risk of thromboembolic events in 1,002 patients with a history of this condition, with an incidence of thromboembolic events of 2.6% in the group without TXA vs. 2.5% in the group with TXA [20]. Wang et al. [14] also reported no differences in the occurrence of thromboembolic events between groups treated with and without TXA among patients undergoing THA and TKA. Poeran et al. [13] reported no association between the use of TXA and increased odds of thromboembolic and ischemic complications among 45,952 high-risk patients who underwent elective lower extremity TJA surgery. Porter et al. [6] reported in their review of 38,220 patients with TJA that the administration of TXA to high-risk patients was not associated with a statistically significant increase in the risk of postoperative complications, including VTE, myocardial infarction (MI), cerebrovascular accident (CVA), death, or readmission at 90 days.

VTE surveillance was symptom-driven; diagnostic imaging was performed only when clinically indicated. Consequently, asymptomatic DVT may have been missed, and the reported incidence reflects symptomatic events only. This approach reflects routine clinical practice in many institutions but differs from that of studies employing systematic postoperative ultrasonographic screening.

Although postoperative hemoglobin and transfusion requirements were prospectively recorded, preoperative hemoglobin, intraoperative blood loss, calculated blood loss, hidden blood loss, and perioperative hemoglobin changes were not systematically collected.

Although the clinical characteristics of the patient with symptomatic VTE provide important context, the descriptive design does not permit conclusions regarding a causal relationship between TXA and thromboembolic risk. Rather, symptomatic VTE was uncommon in patients managed according to the institutional TXA protocol, consistent with previous randomized trials, observational studies, and clinical practice guidelines reporting no increased risk of pulmonary embolism, stroke, or myocardial infarction with TXA use, even in high-risk patients [8]. The principal contribution of this study is to provide prospective real-world data from a Latin American healthcare setting where observational evidence remains limited.

This study had several strengths, including the prospective design, consecutive patient inclusion, complete follow-up, participation of two referral centers, and the reflection of routine clinical practice, providing prospective regional evidence from a Latin American population.

This study also has important limitations. First, the absence of a comparator group precludes the evaluation of the effectiveness or safety of TXA relative to patients managed without antifibrinolytic therapy and prevents causal inference. Second, the number of symptomatic VTE events was very small, limiting the analysis of potential risk factors. Third, symptom-driven VTE surveillance may have underestimated the true incidence of asymptomatic thromboembolic events in this study. Fourth, adherence to each component of the standardized thromboprophylaxis protocol was not prospectively evaluated at the individual patient level; therefore, the potential influence of thromboprophylaxis adherence on postoperative VTE risk could not be analyzed.

These findings should be interpreted as descriptive evidence of routine clinical practice. Nevertheless, real-world studies complement randomized trials by evaluating outcomes under everyday conditions, particularly in healthcare systems underrepresented in the orthopedic literature. Future multicenter studies with comparator cohorts and comprehensive perioperative blood loss assessment are warranted.

## Conclusion

In this prospective, two-center observational cohort study, routine intravenous TXA administration during primary THA, including conventional and complex cases, was associated with a low incidence of symptomatic venous thromboembolism (0.75%) and blood transfusion (3.8%). These findings provide prospective real-world evidence from a Latin American population, contributing regional data on perioperative blood conservation in primary THA. Future multicenter prospective studies including comparator cohorts, larger sample sizes, and comprehensive perioperative blood loss assessment are warranted to further characterize the effectiveness and safety of TXA in routine clinical practice.

## Data Availability

Data associated with this article cannot be disclosed due to ethical reasons.
